# TGF-beta1 Does Not Induce Senescence of Multipotent Mesenchymal Stromal Cells and Has Similar Effects in Early and Late Passages

**DOI:** 10.1371/journal.pone.0077656

**Published:** 2013-10-17

**Authors:** Gudrun Walenda, Khalid Abnaof, Sylvia Joussen, Steffen Meurer, Hubert Smeets, Björn Rath, Kurt Hoffmann, Holger Fröhlich, Martin Zenke, Ralf Weiskirchen, Wolfgang Wagner

**Affiliations:** 1 Helmholtz-Institute for Biomedical Engineering, Stem Cell Biology and Cellular Engineering, RWTH Aachen University Medical School, Aachen, Germany; 2 Algorithmic Bioinformatics, Bonn-Aachen International Center for Information Technology, University of Bonn, Bonn, Germany; 3 Bioanalytical Resource Centre Aachen, Institute for Molecular Biotechnology, RWTH Aachen University, Aachen, Germany; 4 Institute of Clinical Chemistry and Pathobiochemistry, RWTH Aachen University Medical School, Aachen, Germany; 5 Genetics and Molecular Cell Biology, CARIM School for Cardiovascular Diseases, University of Maastricht, Maastricht, Netherlands; 6 Department for Orthopedics, RWTH Aachen University Medical School, Aachen, Germany; 7 Institute for Biomedical Technology, Department of Cell Biology, RWTH Aachen University Medical School, Aachen, Germany; Centro Cardiologico Monzino, Italy

## Abstract

Transforming growth factor-beta 1 (TGF-β1) stimulates a broad range of effects which are cell type dependent, and it has been suggested to induce cellular senescence. On the other hand, long-term culture of multipotent mesenchymal stromal cells (MSCs) has a major impact on their cellular physiology and therefore it is well conceivable that the molecular events triggered by TGF-β1 differ considerably in cells of early and late passages. In this study, we analyzed the effect of TGF-β1 on and during replicative senescence of MSCs. Stimulation with TGF-β1 enhanced proliferation, induced a network like growth pattern and impaired adipogenic and osteogenic differentiation. TGF-β1 did not induce premature senescence. However, due to increased proliferation rates the cells reached replicative senescence earlier than untreated controls. This was also evident, when we analyzed senescence-associated DNA-methylation changes. Gene expression profiles of MSCs differed considerably at relatively early (P 3 - 5) and later passages (P 10). Nonetheless, relative gene expression differences provoked by TGF-β1 at individual time points or in a time course dependent manner (stimulation for 0, 1, 4 and 12 h) were very similar in MSCs of early and late passage. These results support the notion that TGF-β1 has major impact on MSC function, but it does not induce senescence and has similar molecular effects during culture expansion.

## Introduction

Transforming growth factor beta 1 (TGF-β1) triggers complex cellular responses, including activation of SMAD transcription factors, which regulate for example expression of inhibitors of DNA binding proteins 1-3 (ID1, ID2 and ID3) [1]. It has major impact on a multitude of other pathways such as mitogen-activated protein kinase (MAPK), Jun N-terminal kinase (JNK), and the phosphatidylinositol 3-kinase/Akt/mTOR pathways, as well as other down-stream targets of the small GTPases Rho, Rac, and Cdc42 [2–5]. TGF-β1 also up-regulates the cyclin-dependent kinase inhibitors CDKN1A (WAF1; CIP1, p21), CDKN2A (INK4A; p16) and CDKN2B (INK4B; p15) [4,6]. With regard to this variety of implications on the molecular network it may be not surprising that the effects of TGF-β1 are largely dependent on the cell type, the cellular environment and the differentiation state [7,8].

Multipotent mesenchymal stromal cells (MSCs) are concurrently tested in a multitude of clinical trials for a broad range of diseases [9]. They comprise a multipotent subset of cells which is capable of differentiation towards the mesodermal lineages such as adipocytes, osteocytes and chondrocytes [10]. It has been shown that TGF-β is essential for chondrogenic differentiation and supports myogenic differentiation [11,12–12], whereas it negatively effects adipogenic differentiation of MSCs [13,14]. Furthermore, the effect of TGF-β1 on differentiation of MSCs is influenced by substrate elasticity [15,16]. TGF-β alone or in a combination with platelet-derived growth factor (PDGF) and fibroblast growth factor (FGF) was suggested to be required to facilitate *in vitro* proliferation of MSCs [17–19], whereas other studies indicated that it induces cell-cycle arrest in mesodermal cells [20,21]. Some of these contradictory results may be due to the heterogeneous composition of different MSC preparations or culture conditions [22]. 

Even for defined cell preparations and under standardized culture conditions the cellular composition, morphology, and function changes continuously during culture: MSCs - such as all non-transformed primary cells - undergo a process of replicative senescence in the course of culture expansion. After a limited number of cell divisions they unequivocally stop proliferation, acquire a large and flattened cellular morphology, and they lose their *in vitro* differentiation potential [23,24]. These peculiar alterations in cellular physiology are reflected by global gene expression changes [23] and highly reproducible epigenetic modifications. Specific CpG sites in the genome become either hyper- or hypo-methylated upon long-term culture of MSCs [25], and can be used to track the process of cellular aging [26,27]. Thus, it is well conceivable, that effects of TGF-β1 differ considerably in cells of early and later passage. In fact, it has been suggested that the sensitivity towards TGF-β is influenced by the aging process [28–30] and it has been further suggested that this cytokine induces cellular senescence [20,21]. 

In this study, we have further analyzed the effect of TGF-β1 on human bone marrow MSCs, particularly during long-term expansion. Furthermore, we compared the global gene expression changes upon stimulation with TGF-β1 in MSCs of early and late passage to elucidate if the molecular response varies during culture expansion.

## Methods

### Ethics statement

All samples in this study were used after patient’s written consent using guidelines approved by the Ethic Committee of the University of Aachen (Permit number: EK128/09).

### Isolation of MSC from human bone marrow

Multipotent mesenchymal stromal cells were isolated from mononuclear cells (MNCs) by plastic adherence. In brief, bone fragments from caput femoris or tibia plateau from patients undergoing clinical surgery were flushed with phosphate-buffered saline (PBS) and washed twice with PBS. MNC were then resuspended in culture medium consisting of DMEM (1 g/L glucose; PAA Laboratories, Pasching, Austria) supplemented with glutamine, penicillin/streptomycin (both Gibco / life Technologies, UK ) and 10% FSC (PAA) at 37°C in a humidified atmosphere with 5% CO_2_. Medium changes were performed twice per week and MSCs were passaged when reaching 80-90% of confluence. Re-seeding was performed at a density of 10,000 cells/cm^2^.

### Long term cultivation of MSC

To analyze the effect of TGF-β1 on long-term expansion, MSCs of relatively early passage (P1 - P4) were cultured in parallel with or without 1ng/mL recombinant human TGF-β1 (R&D Systems, Inc., Minneapolis, MN 55413 USA) until they reached replicative senescence. After each passage, the cell number was determined using a Neubauer counting chamber (Brand, Wertheim, Germany) and cumulative population doublings (cPD) were calculated as previously described [31].

### Proliferation assay

Cell proliferation was measured with the Thiazolyl Blue Tetrazolium Bromide (MTT) assay as described in our previous work [32]. Briefly, MSCs of passage 3 - 6 were seeded in 96-well plates (3,000 cells/cm^2^) with different concentrations of TGF-β1. After 7 days, cells were washed with PBS and incubated with 1 mM MTT (Sigma Aldrich, St. Louis, MO, USA) for 3.5 hours at 37°C. The excess solution was discarded and formazan crystals were resolved in 4 mM HCl in isopropanol (both from Roth, Karlsruhe, Germany). The absorbance was measured at 590 nm with a reference of 620 nm using a Tecan Infinite 200 plate reader (Tecan Trading, Switzerland). Each measurement included four technical replicas. Alternatively we estimated proliferation by counting of DAPI stained nuclei after 7 d in a 96-well format. Furthermore, we stimulated MSCs with different concentrations of TGF-β1 for 48 h and incubated with BrdU for additional 24h prior to analyzing BrdU incorporation by Cell Proliferation ELISA (Colorimetric; Roche Applied Science, Mannheim, Germany). Anti-BrdU Peroxidase incubation was performed for 90 min and substrate conversion was measured after 5 - 10 min.

### Staining for senescence associated β-galactosidase activity

Activity of pH-dependent senescence-associated β-galactosidase (SA-β-gal) was analyzed simultaneously in different MSC passages using SA-β-gal staining kit (Cell Signaling Technology, Boston, MA, USA). In addition, pH dependent SA-β-gal activity was analyzed with a fluorescence-based method for quantitative and sensitive analysis by flowcytometry [33]. In brief, MSCs were incubated with Bafilomycin A1 (Sigma, St Louis, MO, USA) to prevent lysosomal acidification and subsequently 5-dodecanoylaminofluorescein di-β-D-galactopyranoside (C_12_FDG, Invitrogen, Eugene, OR, USA) was used as a fluorogenic substrate.

### Immunophenotypic analysis

Expression of a panel of surface markers was analyzed in MSCs upon expansion for 4 - 5 passages with or without 1 ng/mL TGF-β1. Cells were stained in parallel with the following monoclonal mouse antihuman antibodies: CD14-APC (clone M5E2), CD29-PE (clone MAR4), CD31-PE (clone WM59), CD34-APC (clone 8G12), CD45-APC (clone HI30), CD73-PE (clone AD2), CD90-APC (clone 5E10) and CD325-PE (clone 8C11; all BD Bioscience, Heidelberg, Germany) and CD105-PE (clone MAR-226; ImmunoTools, Friesoythe, Germany). Analysis was performed using a FACS Canto II cytometer (BD Bioscience) and the collected data were analyzed with WinMDI 2.9 software (The Scripps Institute, Flow Cytometry Core Facility, CA, USA). Fluorescence intensities were normalized to the corresponding autofluorescence measurements for statistical comparison.

### *In vitro* differentiation of MSC

The impact of TGF-β1 on *in vitro* differentiation was analyzed in MSCs which were pre-incubated either with or without 1 ng/mL TGF-β1 for 1 to 4 passages before induction of differentiation and during osteogenic and adipogenic differentiation [34]. After three weeks, calcium-phosphate deposition of osteogenic differentiated cells was analyzed by Alizarin Red stain and formation of fat droplets of adipogenic differentiated cells was analyzed using the green fluorescent dye BODIPY (4,4-difluoro-1,2,5,7,8-pentamethyl-4-bora-3a,4a-diaza-s-indacene; Molecular Probes, Eugene, USA) with DAPI counterstaining of nuclei. Chondrogenic differentiation was induced in pellet culture. The differentiation medium comprises TGF-β and therefore the same differentiation medium was used for TGF-β1-pretreated cells and un-treated controls [34]. After three weeks chondrogenic differentiation was analyzed by Alcian blue and Periodic acid shiff staining according to routine histology protocols and analyzed with a Leica DM IL HC fluorescence microscope (Leica, Wetzlar, Germany)[35]. 

### Senescence-associated DNA-methylation changes

Culture expansion is associated with highly reproducible genomic hyper-methylation and hypo-methylation at specific CpG sites. We analyzed such senescence-associated DNA methylation (SA-DNAm) changes with our recently described Epigentic-Aging-Signature [26,36]. Briefly, DNA was isolated from 2x10^5^ cells with the NucleoSpin Blood kit (Machery Nagel, Düren, Germany) and bisulfite converted. Pyrosequencing of six relevant CpG sites was performed at Varionostic GmbH (Ulm, Germany) as described before [37]. Beta-values (the percentage of DNAm at the respective sites) were then inserted in our online calculator to estimate passage number of cumulative population doublings (http://www.molcell.rwth-aachen.de/dms/).

### Gene expression profiling

MSCs from three different donors at early passage (P3 - P5) and later passage (P10) were used to analyze the kinetic of TGF-β1 stimulation on gene expression. 1x10^6^ cells were seeded into 6-well culture plates and after one day they were stimulated with TGF-β1 at concentrations and periods as indicated in the text. If indicated, we performed serum starvation with two washing steps and incubation for 12h in FCS free culture media before TGF-β1 treatment. RNA was isolated with the miRNeasy kit (Quiagen, Hilden, Germany) and DNAse digestion. RNA concentration and integrity was determined with an Agilent 2100 Bioanalyzer (Agilent Technologies, Inc., Santa Clara, CA, USA). Genome-wide gene expression analysis was then performed using GeneChip Human Gene 1.0 ST Arrays (Affymetrix, Santa Clara, CA, USA). The complete microarray data and additional information have been deposited in NCBIs Gene Expression Omnibus (GEO, http://www.ncbi.nlm.nih.gov/geo/) and are accessible through GEO Series accession number GSE46019. 

### Real time PCR

cDNA synthesis was generated using the High Capacity cDNA Reverse Transcription Kit (Applied Biosystems, Foster City, CA, USA) and real time PCR was performed with the SYBR Green Method. Primers are listed in Table S1 in File S1. Fold change was compared to un-stimulated MSCs and assessed with the ΔΔCt method. 

### Bioinformatics

Analysis of gene expression profiles was performed *via* R and Bioconductor packages [38]. Raw probe intensities were summarized and normalized using Factor Analysis for Robust Microarray Summarization (FARMS) [39]. Differential gene expression analysis was performed for the probe sets, which could be mapped to Entrez gene IDs according to the Bioconductor package “hugenetransclusterst10.db”. Gene expression of late and early cell passages without TGF-β stimulation was compared *via* Linear Models for Microarray Data (Limma) [40] using a factorial design, which takes into account the correlation of the samples along the time course. An FDR of < 5 % was regarded as significant for differential expression [41]. Hierarchical Clustering of genes was performed using Euclidian distance as dissimilarity measure and complete linkage agglomeration. Whole time courses were compared at once using a random-effects model and the empirical Bayes method to estimate probabilities for differential time course expression [42]. A probability of > 0.95 was assumed to indicate differential time-course expression. For further gene set association studies using Kyoto Encyclopedia of Genes and Genomes (KEGG) pathways and Gene Ontology (GO) terms the p-values resulting from a Limma analysis and probabilities resulting from the time-course analysis were taken as ranking scores for genes. Gene sets (KEGG pathways and GO terms) were then tested for their association with these ranking scores by performing a univariate logistic regression [43]. Adjustment of resulting p-values was subsequently done according to Benjamini & Yekutieli’s false discovery rate control under dependency [44]. Significant functional associations to GO terms and KEGG pathways are regarded at FDR ≤ 0.05.

### Statistics

Results are expressed as mean ± standard deviation of independent experiments. The number of biological replica (n) is depicted in the figure legends. Normal distribution of the data was checked using qq-plots and Shapiro-Wilk test. If the data were approximately normally distributed we have estimated the probability of differences using the paired two-sided Student’s T-test. If data were not normally distributed – e.g. due to natural numbers – we used non-parametric Wilcoxon test as indicated in the text. For all tests, p < 0.05 was used as level of significance. 

## Results

### TGF-β1 influences growth pattern and *in vitro* differentiation of MSCs

Culture media for MSCs is usually supplemented with fetal calf serum (FCS) which is contains TGF-β1 - although predominantly in the biologically inactive latent form [45]. Since MSC growth is stalled under serum-free conditions we tested if we could observe similar effects of TGF-β1 in MSCs cultured either with 10% FCS or under serum starvation for 12 h. Under both culture conditions we observed a similar immediate early and transient up-regulation of *ID1*, *ID2* and *ID3* (1 h) followed by a repression of the corresponding genes (4 h) (Figure S1A in File S1) and this is in line with previous studies [1]. Furthermore, we compared different concentrations of TGF-β1 (0, 0.01, 0.1, 0.3, 1, 3, and 10 ng/mL) and the highest up-regulation of *ID1* and *ID3* was observed with 1 ng/mL TGF-β1 (Figure S1B in File S1). Therefore, we decided to perform all subsequent experiments in normal growth medium and with the addition of 1 ng/mL recombinant TGF-β1, if not indicated otherwise.

**Figure 1:**
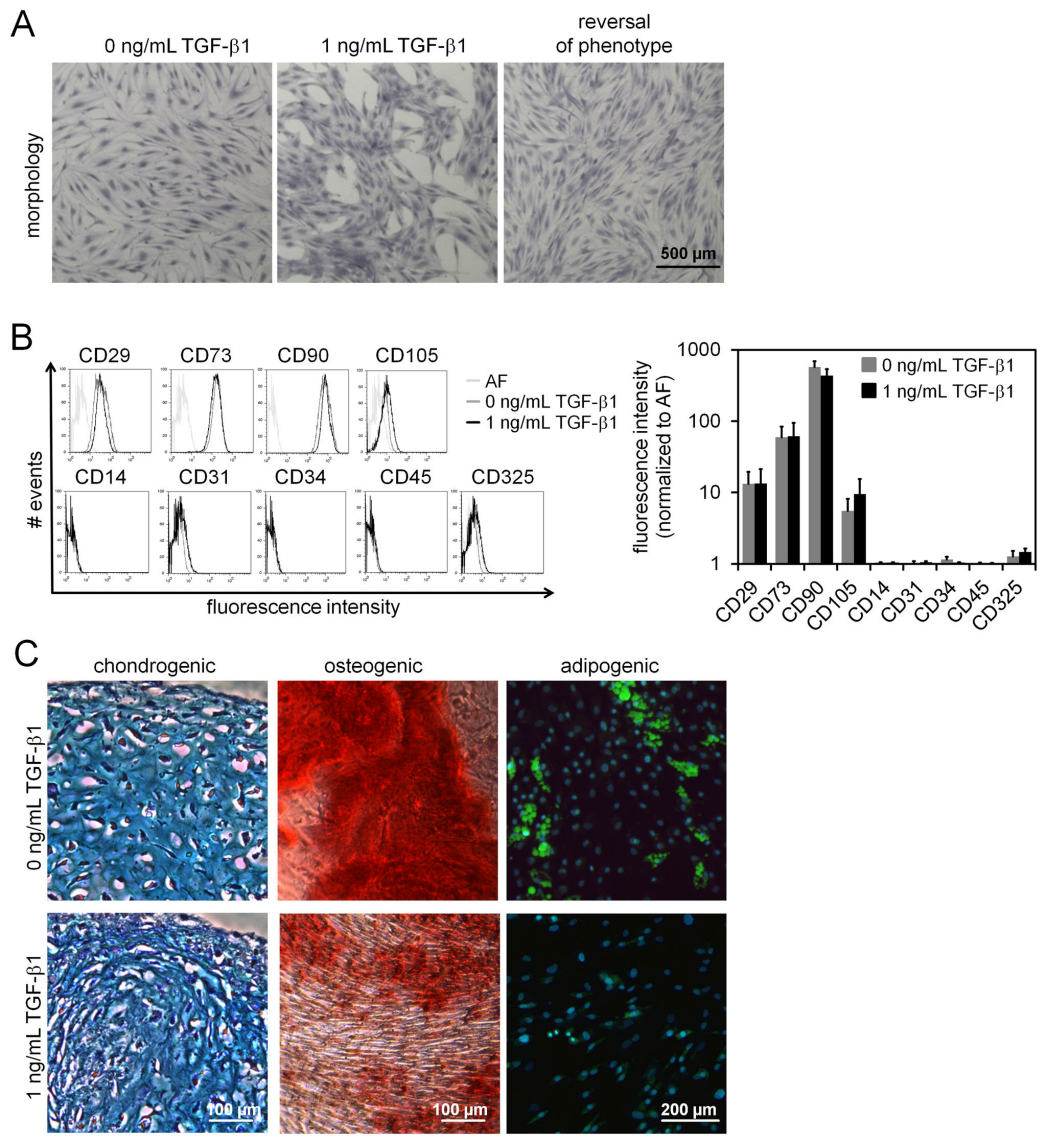
Influence of TGF-β1 on MSC growth and *in* *vitro* differentiation. Treatment with 1 ng/mL TGF-β1 induces a network-like growth pattern of MSCs within 7 days, which is reversed if the cells are re-seeded in media without TGF-β1 (**A**; crystal violet staining of fixed cells). Immunophenotypic analysis of MSCs upon continuous culture either with or without TGF-β1 for 4 to 5 passages was performed by flow cytometry. Exemplary histograms are depicted and analysis of mean fluorescence intensity (normalized to auto-fluorescence) did not reveal significant differences upon treatment with TGF-β1 (**B**; n = 5). MSCs that had been cultured with or without 1 ng/mL TGF-β1 for 1 to 4 passages were differentiated towards chondrogenic, osteogenic and adipogenic lineage. Particularly adipogenic differentiation was impaired by TGF−β1 (**C**; n = 3).

Notably, MSCs cultured with TGF-β1 always revealed a network-like growth pattern whereas untreated cells formed a more homogeneous cellular layer. This effect on growth pattern was reversible when TGF-β1 treated cells were re-seeded in normal culture medium (Figure 1A). All MSC preparations revealed the same typical immunophenotype (CD14^-^, CD29^+^, CD31^-^, CD34^-^, CD45^-^, CD73^+^, CD90^+^, CD105^+^, and CD325^-^), whether they were cultured with or without continuous TGF-β1 stimulation over four passages (Figure 1B). Furthermore, MSCs could be differentiated towards chondrogenic, osteogenic and adipogenic lineage and thus fulfilled standard criteria for definition of MSCs [10]. However, we observed that osteogenic and particularly adipogenic differentiation was greatly impaired if treated with TGF-β1 for several passages and during differentiation (Figure 1C). These results reflect that TGF-β1 has major impact on MSC growth and function.

### TGF-β1 increases proliferation and enhances replicative senescence of MSCs

**Figure 2:**
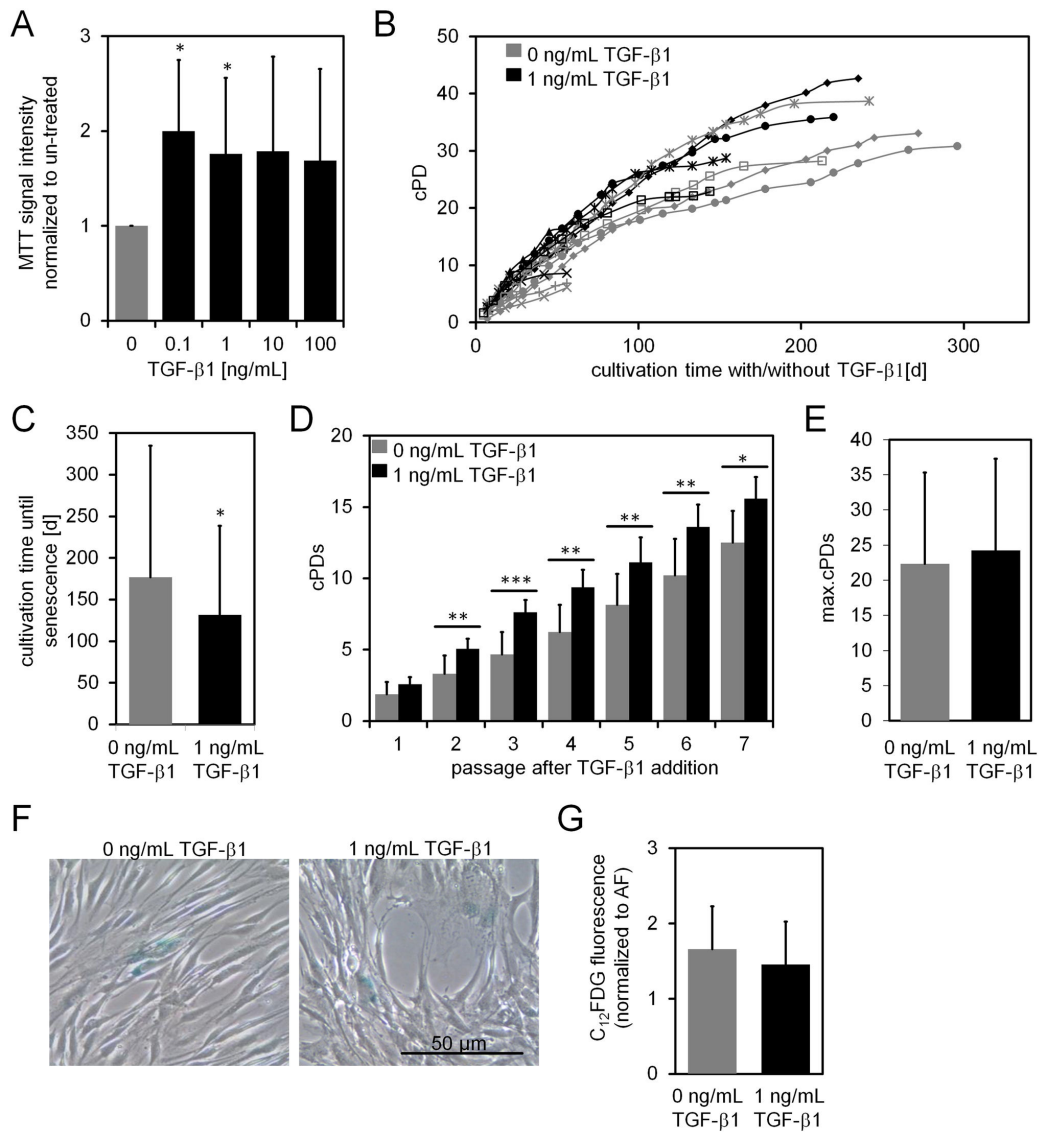
Influence of TGF-β1 on short- and long-term expansion and senescence. MSCs were stimulated for 7 days with increasing concentrations of TGF-β1 and proliferation was estimated using the MTT assay (**A**; n = 5). MSCs were cultured with or without 1 ng/mL TGF-β1 until they stopped proliferation. Cumulative population doublings (cPDs) were calculated throughout culture expansion and depicted by symbols for each passage (**B**; n = 6). The average culture period until proliferation arrest is shorter if cells are continuously cultured with TGF-β1 (**C**; n = 6). Comparison of cPDs for the first seven passages for TGF-β1 treated and un-treated MSC revealed a proliferative advantage particularly in the initial three passages (**D**; n = 6). The maximal cPDs at the time of ultimate proliferation arrest were similar with and without TGF-β1 treatment (**E**; n = 6). SA-β-gal activity was measured in MSCs cultured either with or without TGF-β1 for 7 passages by histochemical analysis with X-gal staining (**E**) or flowcytometric analysis of C_12_FDG (**F**; n = 3; *p < 0.05; **p < 0.01).

To analyze the impact of TGF-β1 on proliferation of MSCs we stimulated the cells with different concentrations of this ligand (0.1 to 100 ng/mL) for seven days and then performed the MTT assay. In comparison to untreated controls proliferation has significantly increased with 0.1 ng/mL (p = 0.018) and with 1 ng/mL (p = 0.05; both Student’s T-test) of TGF-β1 (n = 5; Figure 2A). Similar results were obtained by counting DAPI stained nuclei after seven days of culture (n = 3) or by quantification of BrdU incorporation after three days (n = 6; Figure S2 in File S1). Furthermore, we observed up-regulation of *CDKN2B* after 12 h whereas we did not detect cell cycle arrest in G_0_/G_1_ phase (Figure S3 in File S1). This is in contrast to observations of other groups which demonstrated that TGF-β1 rather impairs proliferation and induces cell cycle arrest in MSCs and fibroblasts [20]. 

Subsequently, we assessed the effect of TGF-β1 on replicative senescence during long-term culture. To this end, we cultured MSCs of early passage in parallel with or without 1 ng/mL TGF-β1 until the cells reached proliferation arrest. Notably, TGF-β1-treated cells entered senescence on average 46 days earlier than untreated controls (p = 0.05; Wilcoxon test; Figure 2C). When we compared cumulative population doublings (cPDs) over each consecutive passage we observed the above mentioned growth promoting effect of TGF-β1, particularly in the initial three passages: supplementation of TGF-β1 increased the number of cPDs by 0.7, 1.73, and 2.94, respectively. In later passages this effect was no more evident, proliferation of non-treated cells slowly caught up until there was no significant difference in the maximal number of cPDs (Student’s T-test; Figure 2D,E). Staining with senescence-associated beta galactosidase (SA-β-Gal), a surrogate marker for senescent cells, did not reveal significant differences after stimulation with TGF-β1 for up to eight weeks (Student’s T-test; Figure 2F,G). 

**Figure 3:**
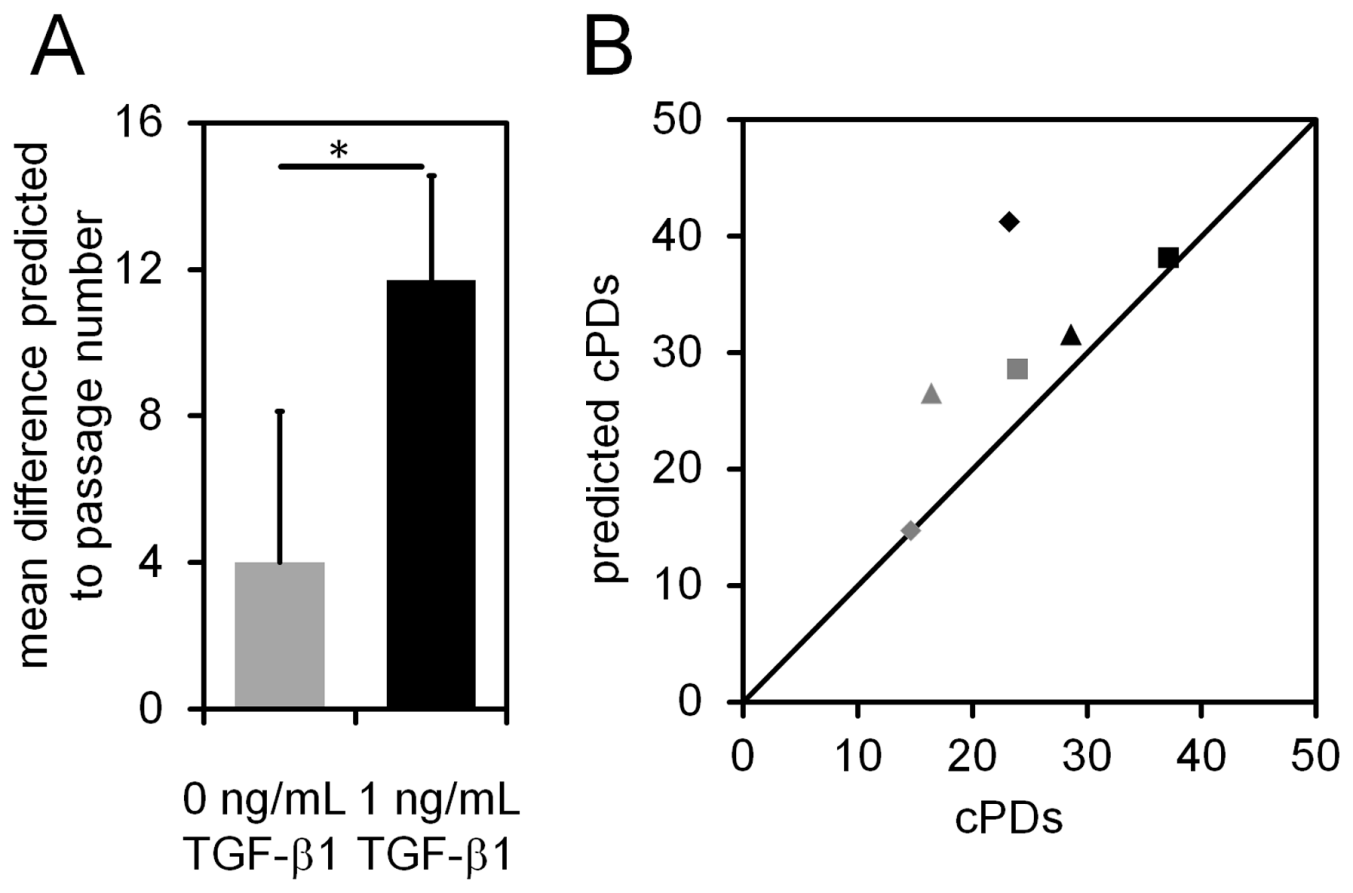
Effect of TGF-β1 on senescence-associated DNA-methylation signature. MSCs were cultured for three passages either with or without TGF-β1. Then the state of cellular senescence was tracked using our recently described Epigenetic-Senescence-Signature which is based on DNA-methylation (DNAm) changes at six specific CpG sites in the genome [35,36]. Predicted passage numbers and real passage numbers correlated well for controls, whereas the passage number was significantly overestimated for TGF-β1 treated cells (**A**; *p < 0.05). Comparison of predicted and real cumulative population doublings (cPDs) reflected the proliferative advantage with TGF-β1. Overall, the number of cPDs was slightly overestimated by the Epigenetic-Aging-Signature but this cannot be attributed to TGF-β1 stimulation (**B**). Symbols represent different MSC preparations that were cultured with (black color) or without TGF-β1 (grey color).

To further analyze if TGF-β1 induces epigenetic senescence-associated changes we used our recently described Epigenetic-Senescence-Signature [26,37]. This method is based on DNA methylation level at six CpG sites and facilitates predictions of passage numbers and cPDs. Cells that were treated with TGF-β1 for five passages were significantly over-estimated in their passage number (p = 0.031; Student’s T-test; Figure 3A), but this can be attributed to the higher number of cell divisions that the cells undergo during this period which is also reflected by the higher numbers of cPDs at the time (Figure 3B). Thus, in relation to the real number of cPDs the predictions of the Epigenetic-Senescence-Signature were not significantly over-estimated. Taken together, TGF-β1 increases proliferation of MSCs and thus they enter replicative senescence after less time. However, we did not observe evidence that TGF-β1 directly induces cellular senescence of MSCs.

### TGF-β1 induces similar gene expression changes in early and late passages

**Figure 4:**
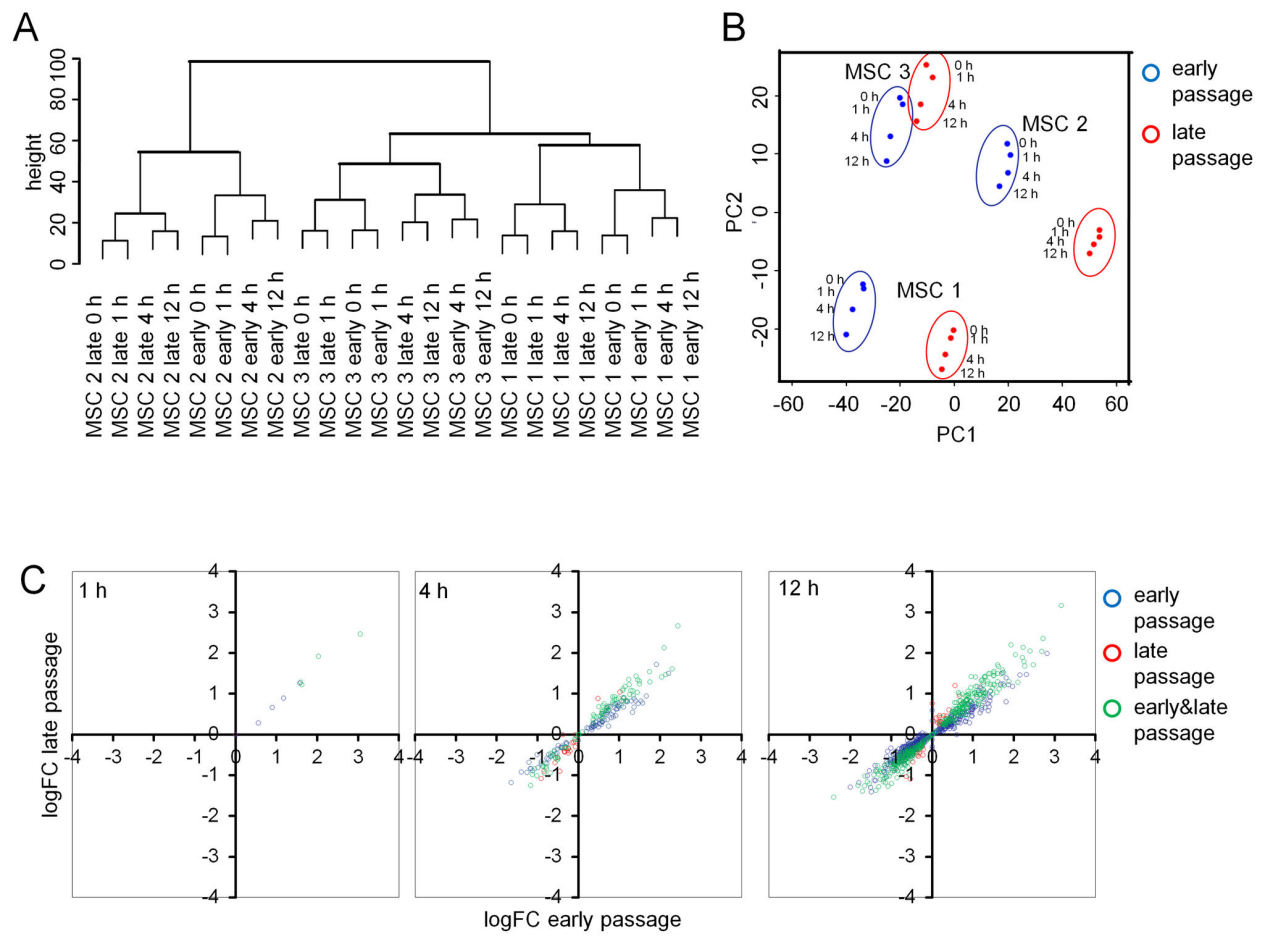
Gene expression changes upon TGF-β1 treatment in early and later passages. Hierarchical clustering of global gene expression profiles (Euclidean distance) revealed inter-donor variation, a close relationship of early and late passages, and continuous changes with TGF-β1 stimulation (**A**). This was also reflected by principal component analysis (**B**; components 1 [PC1] and 2 [PC2] are depicted). TGF-β1-induced gene expression changes were compared in MSCs of early passage (P3 - P5) and later passage (P10) upon stimulation for either 1, 4, or 12 hours. Some genes are predominantly induced in early passage (depicted in blue) or in late passage (depicted in red) but the induced gene expression changes were overall very similar in MSCs of early and later passage (**C**).

Global gene expression changes were analyzed upon stimulation of MSCs with TGF-β1 for 0, 1, 4 and 12 h. These experiments were performed with three MSC preparations at corresponding early (P3 - 5) and later passages (P10). These passage numbers corresponded to cPDs of 6.17 to 7.49 and 14.51 to 16.38, respectively. Microarray data reflected up-regulation of various TGF-β1 response genes, including transient induction of *ID1*, *ID2* and *ID3* which has also been demonstrated by qRT-PCR (Figure S4 in File S1) and is consistent with findings of Kang et al. [46]. Hierarchical clustering of gene expression profiles clearly demonstrated donor-associated variation. Furthermore, corresponding samples of early and late passages clustered together and in tendency gene expression profiles were also sorted according to the time of TGF-β1 stimulation (Figure 4A). These results are also reflected by principal component analysis of the gene expression profiles (Figure 4B). Thus, all three parameters – donor-specificity, passage number, and TGF-β1 – have reproducible impact on gene expression profiles.

Comparison of gene expression profiles of MSCs at early and late passages (all without TGF-β1 stimulation) yielded 345 gene expression changes (File S2). This is in line with our previous work demonstrating that long-term culture of MSCs has major impact on gene expression profiles [23,37,47]. Subsequently, we analyzed differential gene expression upon stimulation with TGF-β1 for either 1, 4, or 12 h in comparison to non-treated controls. The number of differentially expressed genes increased continuously in MSCs of both early and late passages. Notably, there was a very high overlap of TGF-β1 induced genes in early and late passages (Table 1). This became also evident, when we plotted log fold changes in early *versus* late passages (Figure 4C). Noteworthy, the number of senescence-associated genes remained similar throughout the time course (File S2). 

**Table 1:** Number of regulated genes after TGF-β1 treatment.

	**1 h**	**4 h**	**12 h**	**Time course**
**early passage**	10	326	2108	2469
**late passage**	5	336	952	1966
**overlap (early and late passage)**	4	129	646	1285

Numbers of differentially expressed genes after TGF-β1 stimulation compared to untreated MSCs (Limma T-test; FDR < 5%). These changes were either analyzed in MSCs of early passage or MSCs of later passage (gene lists are provided in File S2). The overlap depicts the number of genes which are regulated in early and late passages.

We then analyzed TGF-β1 induced gene expression changes with regard to the whole time course. 2,469 transcripts and 1,966 transcripts revealed continuous gene expression changes in early and late passages, respectively (probability > 95%; Table 1; File S2). The overlap of these time course-associated genes was remarkably high (1,282 genes). When we specifically looked for genes with significantly differential time-courses between early and late passage, only four genes were identified (probability > 95%) – *SULF1, PAQR5, RPLP1*, and *THBS4* – and none of them was more than two-fold differentially expressed upon TGF-β1 stimulation at any time point. 

A follow-up functional analysis for association with KEGG pathways and GO terms revealed very similar TGF-β1 induced gene expression changes in early and late passage (Tables S3 and S4 in File S1). Taken together, our results support the notion that TGF-β1 stimulation has great influence on gene expression in a time course-dependent manner, but there are hardly any differences in TGF-β1 induced gene expression in MSCs of early and late passages. This underlines the similarity of the transcriptional response to TGF-β1 stimulation in early and late passages.

## Discussion

The effects of TGF-β1 are interdependent on a variety of different parameters such as culture media, pretreatment, incubation time and even more critical on cell type and state of differentiation [2]. The goal of this study was to analyze the effect of TGF-β1 in long-term culture of MSCs as it has been suggested that TGF-β1 induces cellular senescence [20]. On the other hand, we assumed that TGF-β1 exerts different gene expression changes in cells of early and late passage. Our results support the notion that TGF-β1 has impact on MSC growth and differentiation but there was no evidence that it induces cellular senescence. Notably, despite the profound alterations in cellular physiology during culture expansion the molecular sequel of TGF-β1 appears to be very similar in cells of early and later passages.

In this study, we describe that TGF-β1 induces a network-like growth pattern in MSCs. It was previously reported that TGF-β induces alterations of the actin-cytoskeleton of MSCs [12,48]. In line with these observations, TGF-β1 induced gene expression was particularly associated with KEGG pathways “regulation of actin cytoskeleton” and “focal adhesion” and to GO-Terms “cell adhesion” and “axon guidance”. Several previous studies have indicated that TGF-β promotes proliferation of MSCs [13,17,19] whereas other authors claimed that it rather induces cell-cycle arrest and even cellular senescence [20,21]. Here, we describe that TGF-β1 significantly enhanced proliferation, particularly if applied in low concentrations. These contradictory results might be due to differences in culture conditions. It may also be attributed to differences between individual MSC preparations which vary based on the different starting material, isolation procedures and even between different donors [22]. 

Adipogenic differentiation was completely inhibited in TGF-β1 pre-cultured MSCs in the presence of TGF-β1 which has been demonstrated in similar studies before [14]. Notably, TGF-β1 treatment also resulted in a significant down-regulation of the peroxisome proliferator-activated receptor gamma (*PPAR-γ*) even without induction of adipogenic differentiation (0.5-fold down-regulation, p = 0.002). *PPAR-γ* is a nuclear hormone receptor which plays a central role in adipogenic differentiation [49]. Thus, down-regulation of this master regulator by TGF-β1 may be one of the reasons for impaired adipogenic differentiation. Osteogenic differentiation was also reduced in TGF-β1 pre-cultured MSCs although this effect was less pronounced and varied between experiments. The osteogenic differentiation marker *RUNX2* was significantly up-regulated by TGF-β1 even without induction medium (2.5-fold up-regulation, p = 0.003). TGF-β1 may stimulate the early stages of osteogenic differentiation whereas it negatively affects subsequent differentiation steps which are characterized by calcium deposition [13,50,51]. Notably, *in vitro* differentiation of MSCs is also affected by long-term culture. Various groups have demonstrated that particularly the adipogenic differentiation decays in long-term culture [23,24,52]. In this regard, there may be a functional association of TGF-β1 stimulation and long-term culture associated changes.

TGF-β1 induced cellular senescence has been discussed as a mechanism to prevent malignant cell transformation into e.g. hepatocellular carcinoma [53] or lymphoma cells [54]. It has also been shown to induce apoptosis or senescence in un-transformed cells, like epithelial or T cells [55,56]. On the other hand, the growth stimulatory effect of TGF-β was discussed to activate malignant or non-malignant cell types, like glioma or smooth muscle cells [57,58]. So far, the senescence-stimulatory effect of TGF-β1 has been particularly detected by cell-cycle analysis and estimation of the SA-β-Gal activity [20]. In this study, we could not observe cell-cycle arrest or signs of premature cellular senescence upon treatment with TGF-β1. 

For clinical applications MSCs are usually used before passage 5 - due to the functional implications of long-term culture and to the risk of malignant transformation [59]. On the other hand, *in vitro* expansion is necessary to obtain enough cells and this applies also to the experiments described in this study. In this regard, the term “early passage” might be misleading – we have used it to discern from MSCs which were expanded for much longer time. We even performed long-term culture experiments with or without addition of TGF-β1 for up to 300 days until the cells reached replicative senescence. 

Some signs of cellular senescence, such as higher prediction of passage numbers with the Epigenetic-Senescence-Signature [26], may be attributed to higher proliferation rates and this may also be the reason for earlier growth arrest with TGF-β1. There was no significant effect on the maximal number of cPDs, or on the predictions of cPDs. These results support the notion, that cPDs are the more appropriate measure for cellular senescence in comparison to passage numbers [60]. TGF-β1 may enhance replicative senescence due to the growth stimulatory effect but it does not directly induce cellular senescence. Usually other growth factors, such as FGF2, bFGF or PDGF-BB are considered to stimulate MSC growth [61,62]. Addition of TGF-β1 to MSC culture media may enhance culture expansion, too [63] – but it interferes with *in vitro* differentiation potential. 

Senescence has major impact on cellular physiology and epigenetics [23] and it has been shown, that epigenetic modifications may lead to alterations in the TGF-β mediated gene expression [64]. Therefore, we anticipated that the signaling cascades of TGF-β1 are also greatly influenced by the state of replicative senescence during culture expansion. So far, only few studies addressed the role of age-related effects on TGF-β signaling in tissues and cells [28–30]. By monitoring the effect on TGF-β1 during cellular senescence it may be possible to better understand age-related effects *in vivo*. As demonstrated by several previous studies, gene expression is greatly affected by TGF-β1 in a time-course dependent manner [65,66]. However, we hardly observed any differences between MSCs of early and later passages. These results indicate that long-term culture associated changes may not be a major parameter for the heterogeneous functions of TGF-β1.

## Supporting Information

File S1 **Combined PDF of Figure S1 - S4, and Tables S1, S3 and S4.** Figure S1: Assessment of TGF-β1 stimulation conditions; Figure S2: TGF-β1 promotes MSC proliferation. Figure S3: TGF-β1 does not induce cell cycle arrest; Figure S4: Validation of microarray data by RT-PCR; Table S1: List of primers that were used for real time PCR; Table S3: KEGG pathways for differential gene expression in time course analysis; Table S4: Changes in gene expression after TGF-β1 stimulation are associated with very similar GO terms in early and late passages.
(PDF)

File S2 **Excel table of significant gene expression changes.** Gene lists are provided for TGF-β1 induction for 1, 4, 12 h; in early and late passages; and at individual time points or throughout the time course. Furthermore, differences between early and late passages are depicted with or without TGF-β1 stimulation.
(XLS)
