# Supplementary material for: TGF-beta1 Does Not Induce Senescence of Multipotent Mesenchymal Stromal Cells and Has Similar Effects in Early and Late Passages
Source: PLoS One. 2013 Oct 17;8(10):e77656. doi: 10.1371/journal.pone.0077656 (PMC3798389; doi:10.1371/journal.pone.0077656)
Supplement: File S1 — Combined PDF of Figure S1 - S4, and Tables S1, S3 and S4. Figure S1: Assessment of TGF-β1 stimulation conditions; Figure S2: TGF-β1 promotes MSC proliferation. Figure S3: TGF-β1 does not induce cell cycle arrest; Figure S4: Validation of microarray data by RT-PCR; Table S1: List of primers that were used for real time PCR; Table S3: KEGG pathways for differential gene expression in time course analysis; Table S4: Changes in gene expression after TGF-β1 stimulation are associated with very similar GO terms in early and late passages. (PDF) [file pone.0077656.s001.pdf]

SUPPORTING INFORMATION 1

TGF-beta1 Does not Induce Senescence of Mesenchymal Stromal Cells and has Similar Effects in Early and Late Passages.

Gudrun Walenda, Khalid Abnaof, Sylvia Joussen, Steffen Meurer, Hubert Smeets, Björn Rath, Kurt Hoffmann, Holger Fröhlich, Martin Zenke, Ralf Weiskirchen and Wolfgang Wagner

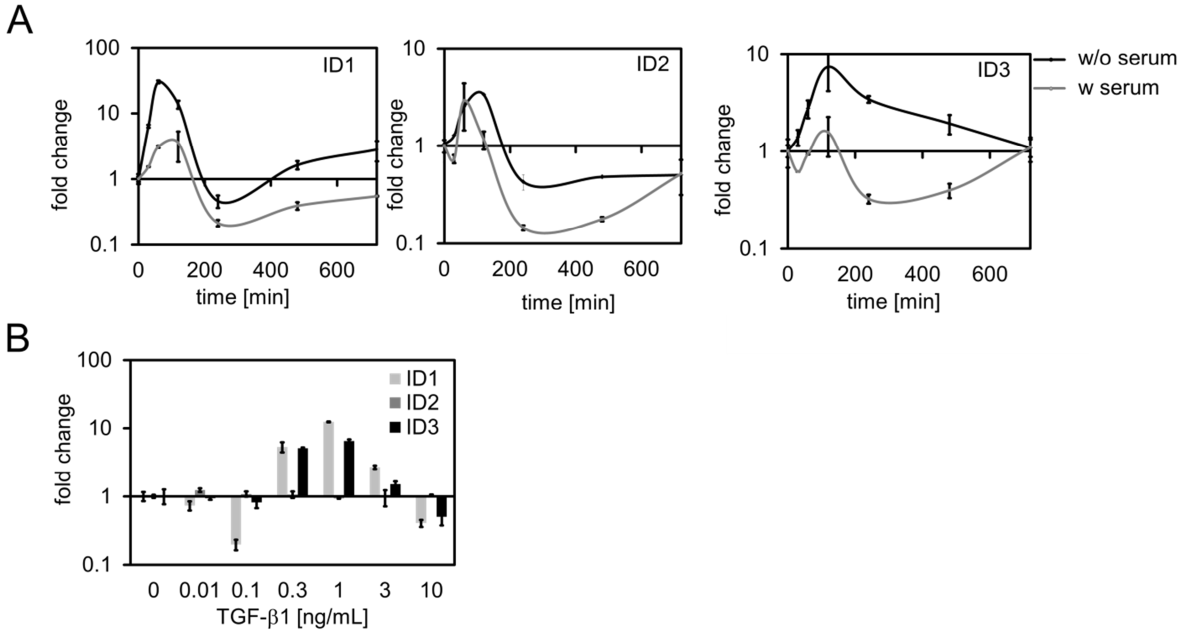

Figure S1. Analysis of kinetics of TGF-β1 stimulation. Preliminary experiments were performed to estimate best stimulation conditions for subsequent experiments. Gene expression analysis of inhibitor of DNA-binding proteins 1, 2, and 3 (*ID1, ID2*, and *ID3*) were tested upon stimulation with 1 ng/mL TGF-β1 (with or without serum supplements) for various time periods **(A)**, and after 1h stimulation with increasing TGF-β1 concentrations **(B)**. Analysis was performed by real time PCR, mean and standard variations are shown for two technical replicas.

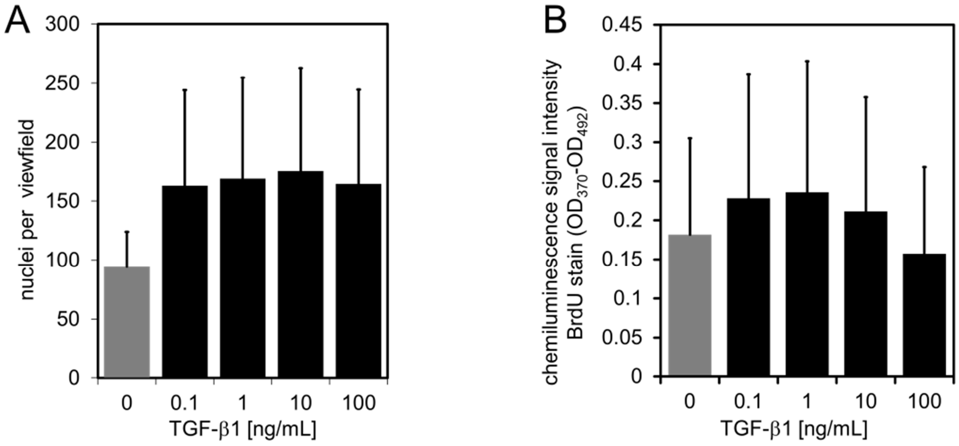

Figure S2. TGF-β1 promotes MSC proliferation. MSC were treated with increasing concentrations of TGF-β1. After 7 d DAPI stained cell nuclei were counted (n = 3; Wilcoxon test; not significant) **(A)**. Alternatively, BrdU incorporation was detected by chemiluminescence signal intensity after 48 h of treatment with increasing concentrations of TGF-β1 (n = 6; Student's T-test; not significant) **(B)**.

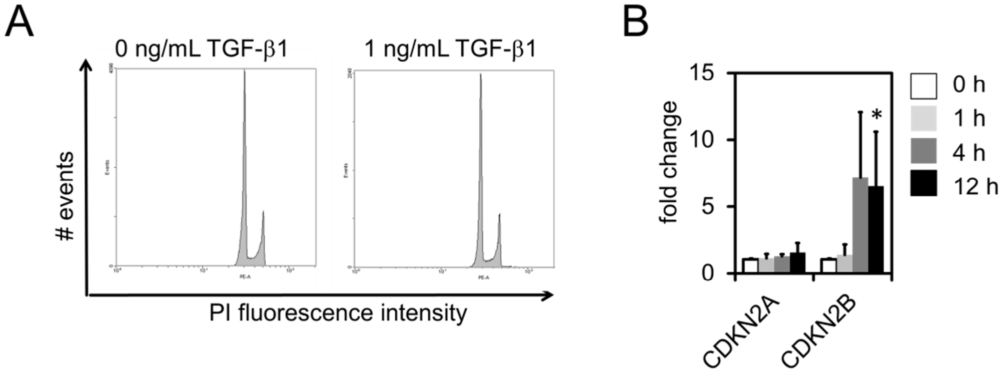

Figure S3. TGF-β1 does not induce cell cycle arrest. Cell cycle analysis by propidium iodide staining was performed upon treatment of MSCs with TGF-β1 for 2 days **(A)**. Gene expression of cell cycle inhibitor *CDKN2B*, but not *CDKN2A* is up-regulated 12 h after TGF-β1 as determined by RT-PCR (n = 5). Mean and standard deviations are shown (*p < 0.05; Student's T-test) **(B)**.

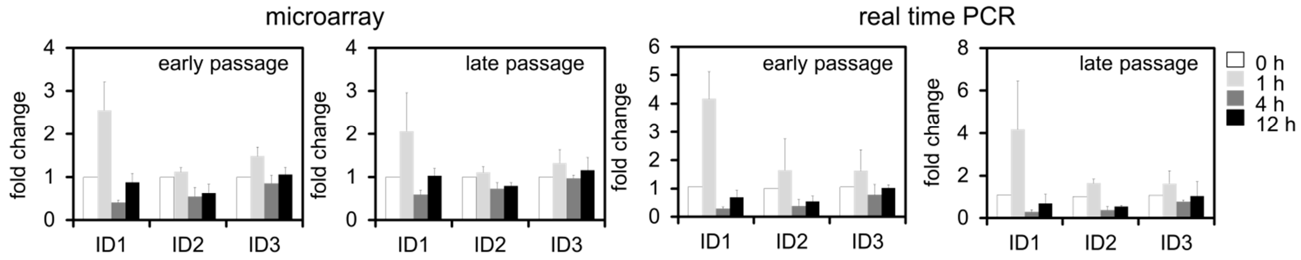

Figure S4. Validation of microarray data by RT-PCR. Gene expression changes of *ID1*, *ID2* and *ID3* were compared in microarray and RT-PCR upon stimulation with 1 ng/mL TGF-β1 for 0, 1, 4, 12 h. Mean fold change and standard deviations are depicted (n = 3).

Table S1. List of primers that were used for real time PCR.

Target	Primer forward	Primer reverse
**ID1**	CCAGAACCGCAAGGTGAG	GGTCCCTGATGTAGTCGATGA
**ID2**	ATATCAGCATCCTGTCCTTGC	AAAGAAATCATGAACACCGCTTA
**ID3**	CATCTCCAACGACAAAAGGAG	CTTCCGGCAGGAGAGGTT
**GAPDH**	TTCGTCATGGGTGTGAACCA	CTGTGGTCATGAGTCCTTCCA
**CDKN2B**	AGTGGAGAAGGTGCGACAG	TCTACATCGGCGATCTAGG
**CDKN2A**	CAACGCACCGAATAGTTACG	AGCACCACCAGCGTGTC

Table S2: Excel table of significant gene expression changes.

Gene lists are provided for TGF-β1 induction for 1, 4, 12 h; in early and late passages; and at individual time points or throughout the time course. Furthermore, differences between early and late passages are depicted with or without TGF-β1 stimulation. This table is not integrated in this PDF and provided as supplemental file 2.

Table S3. KEGG pathways for differential gene expression in time course analysis.

**Early passage**		**Late passage**	
**KEGG Pathway ID**	**FDR**	**KEGG Pathway ID**	**FDR**
05200_Pathways in cancer	3.43E-29	05200_Pathways in cancer	2.77E-26
01100_Metabolic pathways	1.27E-28	01100_Metabolic pathways	5.90E-20
04510_Focal adhesion	4.03E-16	04510_Focal adhesion	3.88E-15
04142_Lysosome	4.03E-16	04810_Regulation of actin cytoskeleton	9.65E-12
04010_MAPK signaling pathway	8.53E-13	04010_MAPK signaling pathway	1.22E-10
04810_Regulation of actin cytoskeleton	2.53E-12	00230_Purine metabolism	5.85E-10
04360_Axon guidance	7.50E-12	04512_ECM-receptor interaction	4.20E-09
04512_ECM-receptor interaction	4.09E-11	04360_Axon guidance	8.81E-09
03030_DNA replication	4.67E-10	05212_Pancreatic cancer	8.81E-09
04350_TGF-beta signaling pathway	1.91E-09	04350_TGF-beta signaling pathway	1.45E-08

Changes in gene expression after TGF-β1 stimulation are mostly associated to the same KEGG pathways in early and late passages (the ten most significant categories are depicted, respectively).

Table S4. Changes in gene expression after TGF-β1 stimulation are associated with very similar GO terms in early and late passages.

Early passage		Late passage	
**GO Term**	**FDR**	**GO Term**	**FDR**
GO:0007165_Signal transduction	1.11E-32	GO:0007165_Signal transduction	4.01E-25
GO:0045944_Positive regulation of transcription from RNA polymerase II promoter	1.11E-32	GO:0045944_Positive regulation of transcription from RNA polymerase II promoter	1.71E-22
GO:0006915_Apoptosis	4.70E-26	GO:0008285_Negative regulation of cell proliferation	7.57E-20
GO:0007275_Multicellular organismal development	1.98E-21	GO:0008284_Positive regulation of cell proliferation	2.80E-19
GO:0008285_Negative regulation of cell proliferation	9.01E-21	GO:0007275_Multicellular organismal development	3.19E-17
GO:0007155_Cell adhesion	2.14E-20	GO:0006915_Apoptosis	3.19E-17
GO:0045893_Positive regulation of transcription DNA-dependent	8.27E-20	GO:0045893_Positive regulation of transcription DNA-dependent	5.65E-17
GO:0043065_Positive regulation of apoptosis	2.71E-19	GO:0007411_Axon guidance	2.25E-16
GO:0008284_Positive regulation of cell proliferation	2.99E-19	GO:0043066_Negative regulation of apoptosis	2.82E-15
GO:0007411_Axon guidance	2.00E-18	GO:0000122_Negative regulation of transcription from RNA polymerase II promoter	3.44E-15

Gene Ontologies for differential gene expression in time course analysis. The top ten categories are depicted.
